# Mapping movie genre evolution (1994 – 2019) using the role of cultural and temporal shifts: a thematic analysis

**DOI:** 10.12688/f1000research.127008.1

**Published:** 2023-06-13

**Authors:** Anshuman Mohanty, Aditi Mudgal, Shirshendu Ganguli

**Affiliations:** 1 Manipal Academy of Higher Education, T A Pai Management Institute, Manipal, Karnataka, 576104, India; 2 Jagdish Sheth School of Management, Bengaluru, Karnataka, India; 3 International Management Institute, Bhubaneshwar, Gothapatna, Bhubaneshwar, Odisha, India

**Keywords:** Movie Genres, Thematic Analysis, Cross Culture Communication Theory, Social Cognitive Theory, and India.

## Abstract

Background: The gratification each person seeks through a movie is different. Sometimes a person would seek information through movies or use them for educational purposes, and some might watch movies to escape into a world of fantasy or humour. Keeping in mind the diverse and ever-changing needs of the audience, the primary objective of this study is to observe the evolution of movie genre and content preference in India, which is one of the largest and culturally intriguing movies producing nation across the globe.

Methods: To attain the objective, the researchers have thematically analysed the top five revenue grossing movie genres over a span of 26 years (1994 to 2019) in Bollywood. More than 100 storylines have been analysed to develop genre trend graphs and the results indicate a sharp decline in the popularity of romantic and family dramas, whereas comedy and action movies have witnessed an overall growth with romantic comedies (romcoms), being the most stable amongst the top five.

Results: Several societal factors like the changing family structure, education level, access to dating applications and even terrorism have been considered to elucidate the evolving psyche of the audience. The theoretical understanding of the result is derived from the uses and gratification theory, cross culture communication theory, and Bandura’s social cognitive theory.

Conclusions: This study would provide  film-makers with a guide to understanding the changing movie genre preferences in India, which in turn would help them to produce economically profitable movies in future.

## Introduction

Movies have been the cornerstone of social and cultural surroundings over the past 100 years. They have not only entertained the viewers but also, they have been a driving force in taking forward political ideologies across generations. Academically, they have enabled us to understand the “society, history and culture” of a country in a lucid way ( Kerrigan, 2017). UNESCO has also recommended preserving movies as they reflect the “cultural identity of people” and as they act as an integral part of the “nation’s cultural heritage” ( Kerrigan, 2017). As movies are consistently mentioned in television, internet and newspapers, they are a common topic for consumer conversations and thus, they have always been instrumental in creating cultural ideologies. This is especially true in India, where the affinity towards movies has always surpassed other entertainment avenues ( Poddar, 2014). The researchers thus focus on the Indian motion picture industry that garnered over $2.1 billion in 2016 and is deemed to be one of the largest movies producing nations in the entire world ( MPAA, 2016).

However, most of the movies released in India are not always profitable. Despite promising numbers, trade reports suggest that around 85% of the movies were not able to recover their investment and labelled as flops in 2016 ( Box office India, 2017). While scrutinizing the top 50 revenue grossers in Bollywood, it was found that the number of profit-making movies reduced from 30 in 2012 to 18 in 2016 and on top of that only 22% of the movies contributed 96% of the overall box office collections. This skewed revenue distribution, along with the short life cycle of movies, coupled by the downward trend in the theatrical sales, poses a serious concern for movie makers and the distributors ( Moon *et al*., 2016). Furthermore, the decline in revenue generation is also attributed to several factors like the increasing rate of piracy, influx of Hollywood movies, shutting down of single screen theatres and so on. However, trade analysts suggest that the most important point of concern for the movie makers is the lack of quality themes and seamless storyline in a movie ( E&Y, 2012). Although marketers and researchers have pondered upon numerous variables like budget, stars, reviews etc. impacting the commercial success of a movie, ‘genre’ is the only element that hints about the probable theme and the storyline ( Zufryden, 1996). As the movie genre captures the audience’s imagination from the word go, it becomes the most decisive factor impacting the success of a movie.

Throughout history there has always been a segregation between commercial movies (genre movies) and art movies (anti-genre ^1^ movies). It is conceded that the major chunk cinemas released in the market are typically genre movies which are “formulaic, conservative and repetitive” in nature ( Jancovich, 2010). Movie genre researcher Barry Keith Grant stated that “…genre movies are those commercial feature films which, through repetition and variation, tell familiar stories with familiar characters in familiar situations” ( Grant, 1995). As genre encapsulates the collective belief of the audience, it is one of the most dominant variables that impacts the box office performance ( Jancovich, 2010; Lee, 2006). Through pre-release buzz and trailers, genre becomes the initial point of contact with the audience, and it inculcates various expectations within them before they select to watch a movie ( Stam and Miller, 2000). As the focus of this study is on the economic aspect of movie making, the researchers have emphasized on the commercial movies released in India.

Commercial movies have always been a dominant force in the Indian market. These movies are popular for its portrayal of musical and well-choreographed dance numbers along with it’s over the top and melodramatic storylines across different genres ( Bouman *et al*., 2010; Mooij, 2006). Although this stereotypical perception of Bollywood holds true for several movies till date, it is also an undeniable fact that genres have continuously reformed their storytelling style over the period of time ( Matusitz and Payano, 2011). Despite the growing importance of movie genres in media studies, scant research has been made on the genre preferences in Bollywood. This absence of literature motivated the researchers to find out are there any evolutionary changes that movie genres and content have gone through in Bollywood over the last two decades?

Thus, the objective of this study is divided into two parts: firstly, the researchers attempt to track the evolution of Bollywood’s most preferred movie genres from 1994 to 2019. Secondly, the researchers investigate the change in the storytelling practices of the most preferred genres over the last 26 years. From a managerial point of view, this research might provide important cues to movie makers in understanding the consumer’s preference and the evolution of movie genres in a better way, guiding them in producing economically profitable movies. As movie watching is deemed to be a cultural phenomenon, the study draws its theoretical standing from the likes of uses & gratification theory ( Weiyan, 2015), cross culture communication theory ( Tannen, 1983), and Bandura’s social cognitive theory ( Bandura, 2005). The theories used in this research, provide a reasoning to comprehend the audience’s behaviour pre, post and during the exposure to movies. The uses & gratification theory (UGT) ( Weiyan, 2015) helps in understanding the mechanism of choosing one genre over the other, which further motivates the content creators to serve the ‘popular’ or ‘in-demand’ content. It is a cyclical process wherein a genre becomes a popular choice among the audience, which brings in commercial success, hence the creators incline towards building stories in and around that genre commonly. The cross-communication theory ( Tannen, 1983) helps one understand the repackaging and adoption of content from other parts of the world and serving them according to the Indian context. The content is flavoured according to the taste preferences of the Indian audiences. The social cognitive theory of Bandura explicates the ongoing evolution of content, i.e., the genre reshaping according to the push and pull of temporal and cultural levers of Indian society. So, a movie reflects and mirrors its society, the storyline brings in the characteristics of the society. It is a two-way process of learning and evolving in forms of sociological and cultural changes which take place continuously. The content reflects the undercurrents a society is going through at a particular time. It becomes a two-way responsibility for both the moviemakers and society to bring fresh ideas, evolve through them and adopt them by making them a part of their life.

## Literature review

The literature review of this study is divided into two parts. Firstly, we throw light on the literature of genre evolution, where we understand that the changes in the kind of movie genres presented can be validated through social cognitive theory and behaviour of choosing a genre over the other, hence making it a popular choice is aligned with Uses and Gratification Theory ( Zia *et al*., 2017). The second section emphasizes on portraying the fact that although several researchers have conducted numerous studies, there is no uniformity in the preference of movie genres across the world. This motivated the researchers to conduct a study focusing on the preference of movie genres in India, which is one of the largest movies producing nations in the world.

### Genre evolution - temporal shifts in content

The temporal fluid nature of the movie genre is the function of the social and cultural taste an audience undergoes over a time frame ( Booth, 2012). This is a gradual change which takes place over the time, an example would be the way consumer behaviour and preferences changes over the due course of time which is mapped in the social cognitive theory ( Schunk, 2012). A movie has the ability to depict a cultural confluence and enact a social change in its storyline. This makes a movie a powerful tool to generate an idea of change in the society and behaviour of the audience ( Gross, 1985). The taste and time are linked and blending both these components help in creating the content for the audience to which they relate and resonate with their real world. The addition of subcultural developments, contextuality, and topical themes of content can be usefully understood as a component of an audience's tastes while choosing a particular genre ( Bourdieu, 1984).

The cultural milieu of societies and the growing globalisation in a cyber era transcends time, distance, and place broadens the scope of cross-cultural amalgamation. The individual choices operate on beliefs, efficacy, sociocentric focus and influence ( Bandura, 1997). These are influenced by the type of futures they seek to achieve collectively as a society. Socio-centric forces interact to re-shape the nature of cultural life, through rapid cultural and technological evolution in their beliefs, morals, social roles, and styles of behaviour. The nature of adaptability and learnability ( Dobzhansky, 1972) helps the human species to remarkably evolve. The humans learn by observing, acquiring knowledge, attitudes, values, emotional proclivities, and competences through the rich fund of information conveyed by actual and symbolic modelling of behaviour in the movies ( Bandura, 1986). The cultures develop and replicate themselves in the form of language, customs, and social practices. In a larger view, one can project the cultural patterns a society has gone through over time.

This agentic capacity rooted in beliefs of personal and collective efficacy to produce effects by one’s actions in a society influence the representation in genre of movies. Cultures are diverse and dynamic social systems which are not static and so is their influence on the current undercurrents integrated with content and influence on genre of movies.

**Figure 1.**
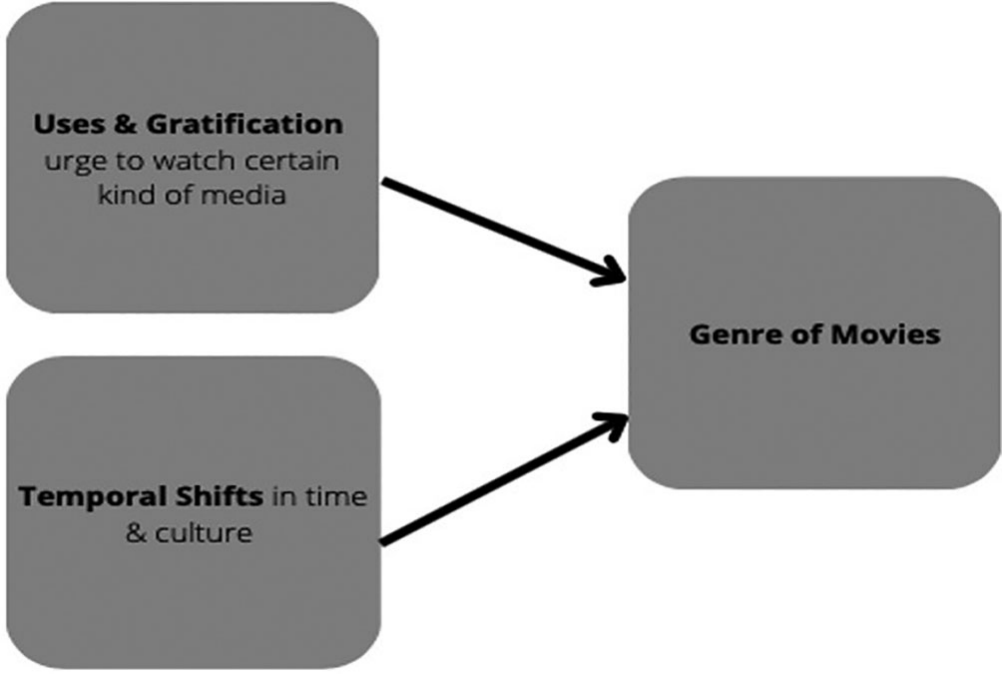
Temporal shift in content (Source: Created by researcher).

Figure 1 helps us in understanding the evolving nature of genres over time. The genre of the movie is dependent on the kind of content satisfying the gratification needs of the audience and the gradual change in the nature and kind of content over the time influenced by subcultural developments taking place in the society. The content is levelled up from time to time to seep in the form of new storylines, genres and plots.

The choice of exposing oneself to a genre of content is personal to everyone. As stated, previous gratification studies conducted in the area of media listed quite a few moderating variables affecting choice. The choice of content is highly dependent on the personal gratification sought by the individual. The repeat behaviour or binge watching a certain kind of content is associated with fulfilment of cognitive and affective gratification needs. The objective of this study is to focus on understanding the evolution of movie genre preferences in India. This study will enable movie makers to identify popular genres that provides the highest gratification to the audience. The theoretical underpinning that supports the process of learning and unlearning behaviour is derived from the social cognitive theory, which explains that a movie genre is temporal and contextual in nature. Movie genres blend in the social and cultural undercurrents to design fresh content for the audience ( Memory, 2008). Thus, movie genres are tweaked according to the changing human behaviour practiced at a particular point of time.

### Movies and genre preference

Traditional motion picture researchers have always tried to generate a proper definition for the movie genre. However, with the passage of time, the idealist thought of defining a genre was discarded away and the overall collective belief of the audience was used to signify the genre for a movie ( Tudor, 1986). Despite Tudor’s thought on genre being widely accepted in the movie literature, a handful of theorists such as Altman (1999) have described that as a movie genre is perceived differently by several user groups, it becomes fairly difficult to come up with a consensus that identifies the true definition of a movie genre. However, Altman’s theory shows very little interest in the consumption process of movie genres. His research on the consumption pattern of a movie genre remained highly abstract and was concerned with several “hypothetical spectators” rather than focusing on the actual behaviour of the society and the market ( Jancovich, 2010). As researchers found it very difficult to find a collectively acceptable definition of genre, several theorists have tried to simplify the situation by distinguishing between the mainstream (genre) versus art (anti-genre) movies. Genre movies are defined as “commercial feature films which, through repetition and variation, tell familiar stories with familiar characters in familiar situations' (Keith, 1995). On the other hand, anti-genre movies are deemed to be free from the formulaic and repetitive storytelling style which is evident in the mainstream movies. As the focus of our research is based on the consumption pattern of top revenue grossing Bollywood movies in India, we focus on the mainstream definition of movie genre.

The relevance of understanding genre preference has been discussed by several researchers in the media literature. The most important aspect of the genre is that it helps the audience in anticipating the prospective storyline of a movie through trailers ( Zufryden, 1996). In the first instance itself, the audiences consider watching or skipping the movie based on their genre preferences. Thus, it becomes very important for the producers to make movies that hits the sweet spot among the audience. This has motivated numerous researchers to conduct studies to comprehend the preference of movie genres in their country. For example, in the USA Brewer *et al*. (2009) analysed the top 100 revenue grossers released between 1997-2001. They reported that although a variety of genres were successful at the box office, violent and adrenaline genres such as horror and action were found to be having more attention seeking power. Meanwhile, Gazley *et al*. (2011) conducted a primary survey in New Zealand and comedies and dramas had more impact on the box office when compared to sci-fi or action movies. In 2012, Walls & McKenzie conducted a study to check whether Hollywood movies portraying American culture dominated the world movie market or whether Hollywood produced movies as per the demand of the world audience. They found out that compared to other genres only Hollywood action movies had a better collection throughout the world. They also pointed out that a successful run in the U.S. market does not guarantee profitable box office returns throughout the world due to cultural disparity. On similar lines Lauren *et al*. (2015) conducted a study on several undergraduates in the U.S. and found that as young Americans had fanciful expectations about romantic relationships, they were more inclined towards romantic drama movies when compared to other genres.

In China, Ye and Starkey (2018) were involved in a study in which they had to ascertain the diversity in genre preference between East Asian nations and countries belonging to Central Eastern Europe. Their study reported that the Asian nations were more inclined towards action-adventure movies compared to the European nations who displayed a higher preference for romantic and comedy genres. Simon *et al*., (2019) conducted a similar study to analyze the relationship between adolescents and their movie watching preferences in eight different countries in Europe. They reported that comedy and adventure were the most popular genres and the preference for suspense, sci-fi and romance varied across countries. Nagaraj *et al*. (2019) analysed the Malaysian audience to determine the reason behind the degrading demand of Malaysian movies when compared to Hollywood. Their results depicted that the Malaysian audience had a higher preference for Hollywood because of their affinity towards action and horror genre movies. As Malaysian movies were not able to cater their needs, the audience shifted towards Hollywood movies. Jam *et al*. (2020) surveyed 400 respondents to study the preference of movie genres in Pakistan. Interestingly they reported that around 80% of the audiences were inclined towards patriotic movies as they were proud of their armed forces and such movies ignited a sense of eternal love for their country.

## Methods

For the first objective, the researchers collected secondary data consisting of the top five revenue grossing movies released between 1994 to 2019. The data was collected from boxofficeindia.com, as it is regarded to be one of the most important data repositories of Bollywood movies in India. The researchers opted for the top five leading revenue grossers as they indicate the highest number of footfalls the movie attracted in a particular year. The researchers generated a comprehensive list of 130 movies divided into twelve genres (Alien, Superhero, Action, Comedy, Dance, Family Drama, Horror, Mythology, Rom – Com, Romantic Drama, Sports Drama and Thriller). However, for the analysis the researchers opted to select only the top 5 recurring genres in the list (Action, Comedy, Romantic Comedy, Family Drama and Romantic Drama) which consisted of 105 (81%) out of the 130 movies (see Table 1). The remaining 25 movies were not included in the analysis as they were not sufficient to form a trend line for the analysis. For the second objective, that is to comprehend the evolution of Bollywood’s most preferred movie genres the researchers have adopted the approach by Longart *et al*., (2018) of generating themes by conducting thematic analysis (TA) of the storyline of 105 movies. Braun and Clarke (2012) defined TA as “A method for systematically identifying, organizing and offering insight into patterns of meaning (themes) across a dataset”. TA helps the researcher to come up with meaningful and sensible patterns from an available set of texts. The history of TA in media and advertising literature dates back to the 1970s ( Barcus,1980). In recent years, Nelson and Salawu (2016) implemented TA to analyse the impact of covert product placements in Spiderman movies. Similarly in Britain, Cranwell *et al*. (2017) conducted TA to study the impact of alcohol abuse and sexual objectification in YouTube videos.

**Table 1.** Total number of movies.

Genre	Number of films
Romantic drama	30
Action	29
Family drama	17
Comedy	15
Romantic comedies	14
Total	105

### Analysis and results

**Figure 2.**
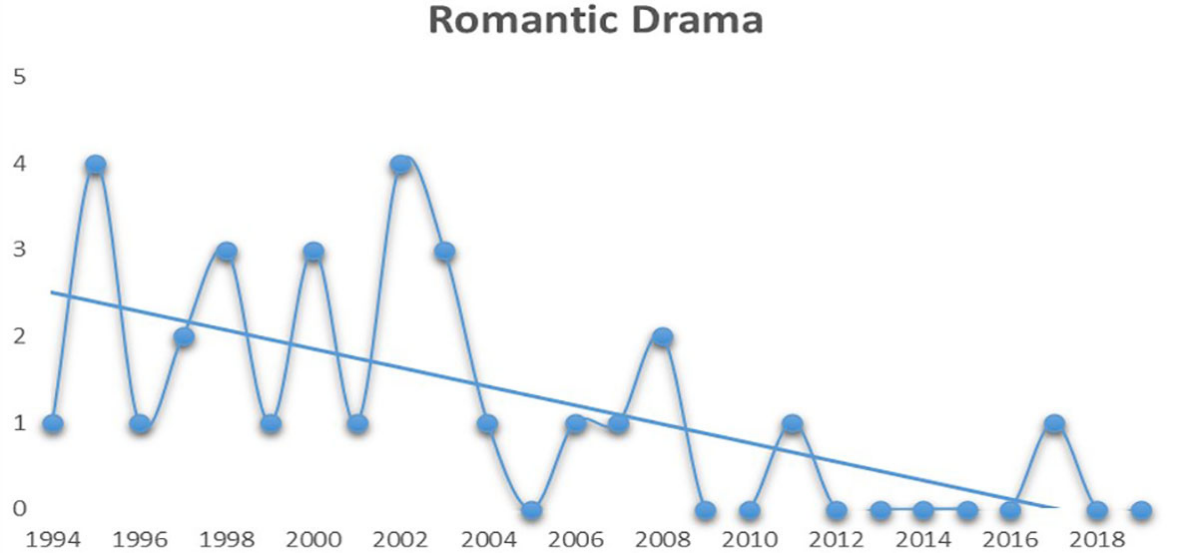
Romantic drama (Source: Created by researcher).

To address the first objective of the study the researchers have considered analysing the trend of the top five revenue grossing genres between 1994 to 2019. A total of 105 movies are spread across the five genres. The genre with the highest frequency of movies is Romantic Dramas followed by Action, Family Drama, Comedy and Romantic Comedies. Figure 2 displays the trend of Romantic Dramas in Bollywood.

**Figure 3.**
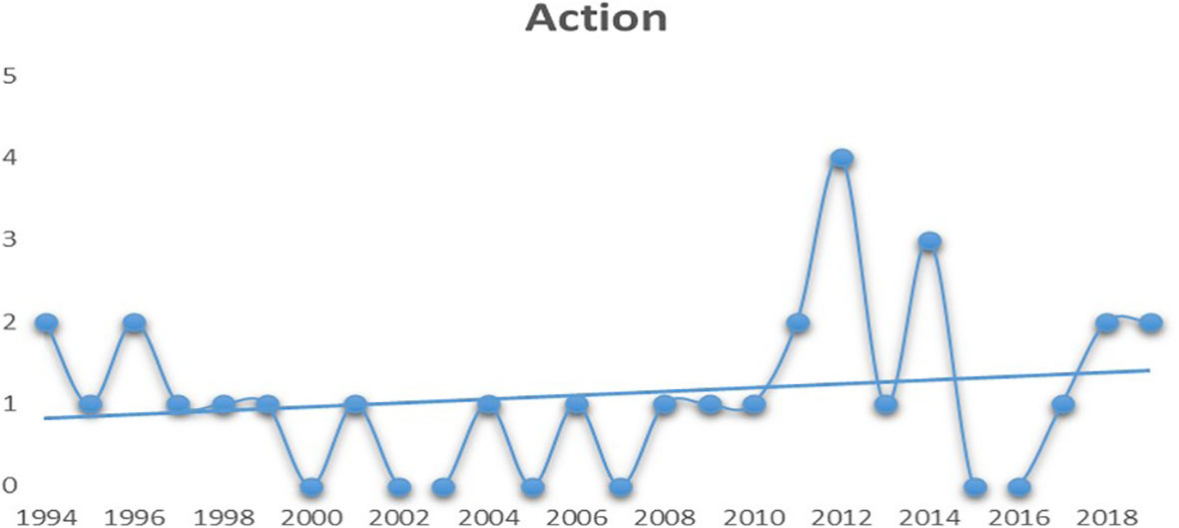
Action (Source: Created by researcher).

From Figure 2 it can be comprehended that the frequency of romantic dramas being commercially successful has drastically reduced after the early 2000s as compared to Action movies (see Figure 3), which has seen a steady growth after the early 2000s.

**Figure 4.**
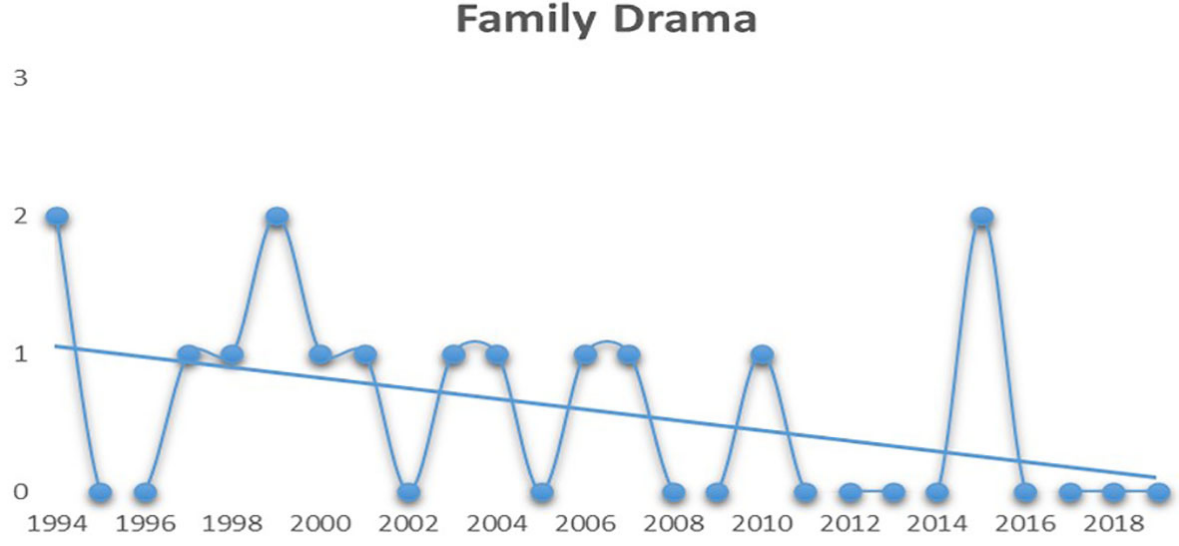
Family drama (Source: Created by researcher).

As depicted in Figure 4, it can be interpreted that the audiences have also started to lose interest in family dramas post 2010. Although the graph displays a huge spike in 2015, one should notice that only two mainstream family drama movies have managed to hit the top five spots over the last eight years.

**Figure 5.**
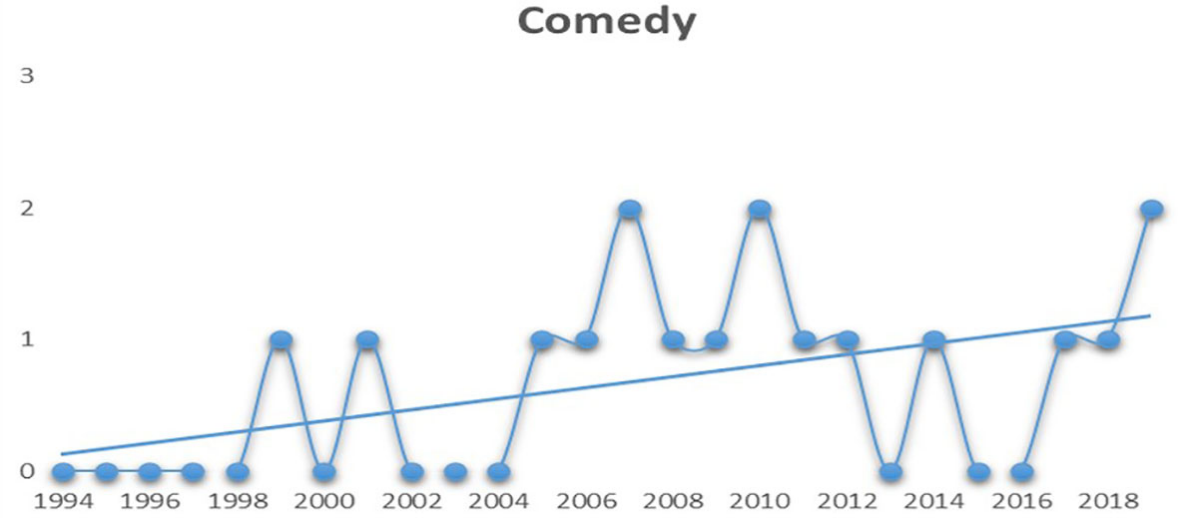
Comedy (Source: Created by researcher).

When compared to the remaining genres, comedy films have managed to hold a steady graph after 2004 (see Figure 5). Except for the years 2013 and 2016, they have always secured a spot among the top five revenue grossers.

**Figure 6.**
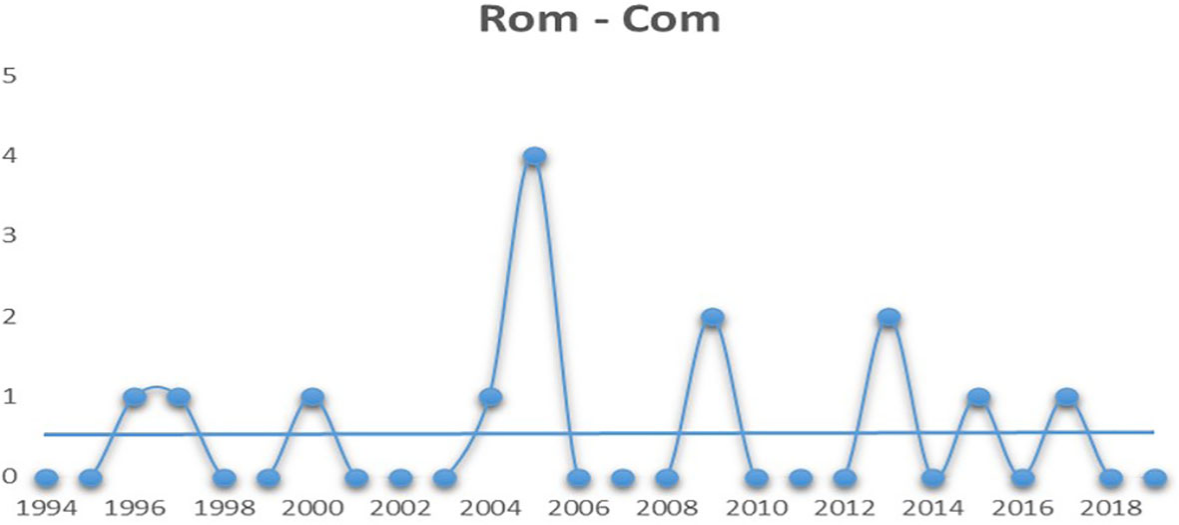
Romantic comedy (Source: Created by researcher).

The final genre on the list is romantic comedies. It is deemed to be a hybrid genre as it is an amalgamation of comedy and romance. Romantic comedies are typically light-hearted, humorous stories which revolves around romantic ideals such as ‘true love’ ( Filmbug, 2021). From Figure 6 it can be seen that although not as steady as comedy movies, romantic comedies have managed to pull off a balanced run over the last few years.

**Figure 7.**
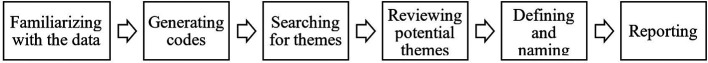
Six phase approach of Thematic Analysis (Adapted from Braun and Clarke 2006).

For addressing the second objective, the researchers extracted the storyline (in text format) of 105 movies from boxofficeindia.comand imdb.com to conduct the TA. The data corpus consisted of 27,572 words, divided into five different genres. To recognize a specific pattern the researchers conducted TA on the textual data and generated several codes to understand them. The analysis follows the six- phase approach of TA (see Figure 7).

The first phase of the TA was to familiarize the researcher with the dataset. The researchers went through the dataset more than twice in order to highlight important points in the text for future analysis. Once the researchers were familiarized with the dataset, they manually coded the data in alignment with the research objectives. One should take into account that codes are not fully written up explanations, rather they are compact and small descriptions of the underlying text. The researchers followed a deductive method of coding. The deductive method of coding is used when the researcher is investigating a specific question and is anticipating a detailed and explicit description for only one specific aspect of the data ( Boyatzis, 1998). To check the stability of the codes the researchers coded the transcriptions more than once. However, to improve the reproducibility of the codes, two external coders (having prior coding experience and keen interest in Bollywood films) were selected for the process. To avoid any misinterpretation, the coders were provided with instructions which clearly mentioned the objective and scope of the study along with the sampling procedure which helped the coder to set boundaries while coding. Finally, a total of 24 codes and 10 themes were extracted from the dataset which was spread across the five genres (see Table 2).

**Table 2.** Codes and Themes derived from movie storylines

Genre	Themes	Excerpts	Codes
Romantic drama	Restricted access to romantic relationships (Early 90s)	He is in love with Mili but is too scared to tell her because knowing how ambitious Mili is, he fears rejection.	Not able to express love
		Sumitra publicly announces her desire for Devdas and Paro to marry, and Kaushalya rejects and humiliates her in public by saying that she is from a lower-class family.	Economic disparity
		Sumitra hastily arranges Paro's marriage to a man from a family which is wealthier than the Mukherjee family: Thakur Bhuvan Chaudhry, a forty-year-old widower aristocrat with three grown children	Arrange and forceful marriages of the female lead (Strict family members)
		Aditya's rich lifestyle and Suhani's middle-class lifestyle creates a rift between their parents. Aditya and Suhani elope and get married in a small ceremony.	Elope and marry
	Realistic storylines (After 2000s)	Aditya and Suhani then move to a dilapidated house and start their married life together.	Marital problems
		Soon, marital problems threaten to drive them apart.	
		He makes a couple of temporary adjustments to solve the problem, first taking her to a neighbour's house which has a portable toilet for a bedridden elderly woman, and later in a train that has a seven-minute stop at the village railway station	Societal problems
Family drama	Emotional conflicts (1990s – early 2000)	He spontaneously decides to marry her despite his father's hostility. When he brings Anjali home, Yash disowns Rahul, reminding him of his adopted status	Economic disparity
		After a futile suicide attempt, Nandini reluctantly weds Vanraj.	Arrange and forceful marriages
		Raja decides to be a faithful, responsible son and vows to meet his parents every wish; including the arranged marriage	Moral and family values
	Addressing Societal problems (After early 2000)	Nikumbh returns and subsequently brings up the topic of dyslexia in a class by offering a list of famous people who were dyslexic	Addressing sensitive issues
		After rescuing her (from a brothel), he vows to take Munni home (Pakistan) on his own despite not having a passport and visa.	Empathy for others
Action	Family revenge stories (1990s – early 2000s)	Karan Arjun, and return to their mother Durga to take revenge upon Durjan Singh.	Family revenge stories
	Addressing national threats (Late 2000s)	During a dark night in Iraq, an American journalist types a warning message to CIA before he is slaughtered by some ISC guards.	Terrorism
		a Deputy Commissioner of Police, gets transferred to Mumbai and discovers that one of his team members, Mahesh, is found dead inside an ambulance with massive bags of money	Corrupt system
		It shows how guns and drugs are smuggled in across the border of Rajasthan, and reach the interiors of India.	Illegal activities
	Female Empowerment (Late 2000s)	They go to the hospital while Zoya leaves Tiger to first kill Baghdavi and his troops which she does with the help of some Syrian girls.	Heroine and hero work together
		ACP Shonali Bose, Jai's college mate, now a police officer in her own right. For the last two years Shonali has been tracking these amazing thefts and is now an expert on this thief, who no one has seen.	Strong female character
Comedy	Rise of mainstream slapstick comedies (After early 2000s)	The sperm samples get mixed up, Deepti happens to be carrying Honey's child and Monika is carrying Varun's child.	Confusion
		Three robbers Pappi, Daga and Teja rob the queen's necklace and are on the run from police and end up in Goa. Pappi, the don, who suffers from short-term memory loss, hides the necklace in Pritam's house.	Funny Gangsters
		The story is based on reincarnation, spanning a period of 600 years from 1419 to 2019. Harry is a barber who frequently gets bizarre flashes of a past era. Harry's forgetful nature causes him to misplace a bag of 5 million pounds which belonged to a gangster named Michael Bhai.	Absurd situations
Romantic comedy	Liberated lifestyle (After early 2000s)	"Nick" "Arora and Ambar" "Amby" Malhotra are two progressive, young Indians who have left India to live in Melbourne, Australia.	Free spirited characters
		He is married to Pooja (Esha Deol), who is very trusting, even though he has been having several affairs with numerous gorgeous women.	Dating life
	Individual aspirations (After early 2000s)	They are happy with each other, but do not believe in tying each other down, so when career beckons they have a mutual break-up but remain friends.	Career oriented
		Bunny is a charmer whose dream is to wander and discover the world, and has no interest of ever settling down.	Following passion

Based on the storyline of the movies, the researchers divided the overarching theme of romantic dramas into two broad domains. Firstly, ‘Restricted access to romantic relationships which was prevalent during the early 90s’ and secondly ‘Realistic portrayal of romantic relationships after 2000’. Similarly, the two overarching themes for family dramas were ‘addressing emotional conflicts during the 90s and early 2000s’ which gradually shifted their focus towards ‘addressing societal problems after the early 2000s’. Action movies were classified into three themes. Predominantly, ‘family revenge stories were common during the early 90s and early 2000s’ which slowly transformed towards addressing ‘national security threats after early 2000s’. Another theme that emerged with the on-set of the millennium was more focus on ‘female empowerment’ and strong female characters in action movies. While analysing comedy movies, the researchers found that the popularity of ‘slapstick comedies’ surged during the 2000s and is still going strong till date. And finally, the researchers attributed two broad themes with romantic comedies. Although there were a few hit romantic comedies in the early 90s, the major change was visible with the dawn of the twenty-first century. The two primary themes attributed with romantic comedies were their focus on ‘liberated lifestyle’ and ‘individual aspirations’ after the early 2000s.

## Discussion

### Romantic drama

Theme 1: Restricted access to romantic relationships (early 1990s)

Culturally, India has been a country which is associated with arranged marriages. Although the Indian literature and scriptures reported the importance of love and romance in abundance, yet romantic relationships have not been a decisive factor in prospective marital alliance ( Gupta 1976). The prospect of a romantic relationship attracted social embarrassment and thus, despite their displeasure young individuals in India abstained from getting involved and expressing their love romantically (Bhalerao, 2018). Thus, the researchers theorize that most of the young individuals were deprived of romantic relationships, and they imagined themselves in the shoes of the main character and momentarily escaped reality by seeking out emotional closure from romantic movies. This made romantic dramas extremely popular in India. However, there was a drop in the popularity of romantic dramas over the last two decades as the Indian population started opening up about romantic relationships explicitly. With access to the internet and Hollywood content during the early 2000s, the Indian population was highly influenced by the liberal and independent lifestyle of the West.

One of the most evident features of the western civilization was the access to unrestricted “partner selection and relationship maintenance” (Bejanyan, 2015). As the modern Indian population was exposed to the western culture, the concept of dating evolved in the country which resulted in the growth of dating applications and love marriages (Gogaga, 2018). The process of selecting a life partner underwent a change in India. The decision of choosing their life-partners also became more of a personal choice than a family discussion ( Sarkar and Rizzi, 2020). Thus, the researchers point out that, as the younger population found romantic closure through online dating and unobtrusive matchmaking in their workplace, they gradually distanced themselves from the highly redundant and melodramatic content of romantic movies (Gittel, 2017). This social change was reflected in the way the number of family dramas and romantic drama movies was reduced. The romantic movies were shifting to exposure and inclusion of liberal values and excluding family storylines out of them, making them unfit for family movie night affairs.

Theme 2: Realistic storylines (after 2000s)

Most of the Bollywood romantic movies in the 90s like Dilwale dulhaniya le jaenge (1995), Pardes (1997), Pyaar to hona hi tha (1998) etc. ended on a happy note, where the male and the female lead would get married and live happily ever after. However, with the decline in the popularity of such romantic movies during the early 2000s, the movie makers sought to reformulate the over-the-top melodramatic content of romantic movies by incorporating realistic storylines (Chakraborty, 2018). They started reflecting on the post marital disagreements between couples, which is a very common occurrence in India (TOI, 2018). Culture and communication go hand in hand, the cross-culture communication theory ( Tannen, 1983) helps us in understanding the phenomenon where the Indian audience shifted from positive stories to realistic stories and chased a practical slice of life content. This kind of content enables them to resonate more with their own life struggles, where each day might not end on a happy note. Thus, the young population of India have shown an inclination towards accepting realistic love stories which tends to capture the bitter and sweet aspects of a modern-day relationship (Filmfare, 2021). As people were becoming more aware of the societal problems in India, romantic movies like Hamara Dil Aapke Paas Hai (2000) and Toilet – Ek Prem Katha (2017) also became very popular as they addressed societal problems like rape and the importance of hygiene in the purview of a romantic backdrop ( Jhunjhunwala, 2020). Thus, to keep the romance genre alive the movie makers transformed the content of Bollywood romantic dramas making them more realistic and relevant for the audience.

### Family drama

Theme 1: Emotional Conflicts (1990s – early 2000)

This theme categorizes family movies that focus on the ego clash between the rich and the poor, intra family conflicts and moral dilemmas. These storytelling attributes were common till the early 2000s. The presence of a joint family was very common in all these movies. The primary content of these movies focused on the clash between diverse economic backgrounds, suppression of the female lead and demonstrating strong Indian family values. However, the popularity of family dramas saw a drastic fall after the early 2000s and their story telling style also transformed. The most visible change was in portrayal of an Indian family structure in a movie during the early 2000s. Researchers have pointed out that the traditional Indian family structure was predominantly based on the joint family culture for centuries ( Sinha, 1984). However, urbanization and rapid industrialization impacted the society drastically by altering the employment scenario in the country. As most of the Indian population was slowly shifting from the joint family structure to a nuclear family structure, their taste in the family dramas also transformed over time. Opening up of Indian society in terms of family structure from joint to nuclear family structure brought in the concept of individual decision making. These changes were captured in the family drama movies as well. For example, most of the successful family drama movies released during the 90s and early 2000s such as Hum aapke hain kaun (1994), Hum saath saath hain (1999) and Kabhi khusi kabhi gham (2001) depicted the conflicts faced within a joint family. However, with time Bollywood family dramas like Taare zameen par (2008) and My name is Khan (2010) started portraying the problems confronted in a nuclear family.

Theme 2: Addressing Societal Problems: (After early 2000)

With time, the family drama movies not only shifted their focus from joint to nuclear families, but they also started addressing societal and sensitive problems like child education, religious harmony and illegitimate childbirth. Movies like My name is Khan (2010), Kya Kehna (2000) and Taare Zameen Par (2008) touched upon sensitive topics with a strong focus on nuclear family values. The audience found themselves more connected to stories which focused on the problems faced by a nuclear family due to the change in the overall family structure in the country ( Jhambekar, 2015). Taking a lesson from the past, the movie-makers intentionally brought in the element of our contemporary culture with an amalgamation of social message to serve different needs of the society ( Tannen, 1983). This reflected the changes the Indian society underwent at different times and contexts. The movies preach and bring the culture of acceptance of new reformative steps the society witnessed as a part of entertainment with fresh genres of movies and sometimes mixing two genres together.

### Action

Theme 1: Family revenge stories (1990s – early 2000s)

Most of the popular blockbusters released in the 90s and 2000s like Karan Arjun (1995), Ghatak (1996) Soldier (1998) and Gadar (2001) were typically family revenge stories. However, with time this trend slowly changed, and family/romance driven revenge stories took a back seat after the early 2000s. The moviemakers realized that the audiences were not interested in watching family driven action stories and thus, they reformulated their story-telling style. Furthermore, after the growth of the internet and influx of English movie channels via cable television during the early 2000s, the Indian audience was exposed to several Hollywood movies which also had an impact on their movie watching preference. As explained in the previous section, the taste of the audience was slowly shifting from family driven movies during the 2000s which had a spill over effect on the Action genre as well. This movie genre was completely influenced by western standards of sci-fi and action movies. The west was a step ahead in introducing this genre. The exposure to such content increased the motivation of movie makers and seekers both. Hence, we see a change in preference of audiences shifting movies towards action, thrillers and scientific thinking.

Theme 2: Addressing national threats (Late 2000s)

The most popular Bollywood action blockbusters released in early 2000s like Dhoom (2004), Dhoom 2 (2007) and Ghajini (2008) were heavily inspired by Hollywood flicks. However, there was a drastic change in the content of Bollywood action movies after the 26/11 Taj Attack at Mumbai. A similar type of paradigm shift was also visible in Hollywood after the 9/11 World Trade Centre attack in New York. The world saw a rise of superhero movies being released in Hollywood, starting with Spiderman in May 2002. As America was grappling with immense grief, Hollywood’s response to this unjust tragedy was felt by the continuous release of superhero movies which instilled a sense of hope and reassurance among them (Hsiao, 2016). Based on the existing literature, the researchers of this study theorize that a similar course of events unfolded in Bollywood after the 26/11 attack in Taj at Mumbai. As the entire population was feeling insecure about the safety conditions in the country, Bollywood’s response to this scenario was to produce movies which presented the heroics of the Mumbai Police and the Indian Army. Movies like Wanted (2009), Dabangg (2010), Singham (2011), Ek Tha Tiger (2012), Tiger Zinda Hai (2017), Simmba (2018), URI (2019) and many others were released, where the main protagonists worked for the police or the army and fought against terrorist organizations, corrupt officers, drug abuse, human trafficking etc. Like many other developing nations, India also faced several threats and Bollywood’s response was to produce movies which instil a sense of faith and confidence in the Indian security forces. Thus, this study theorizes that as most of these action flicks tend to develop stories showcasing real national threats, it evokes a sense of patriotism and valour among the audience and hence they tend to feel more connected with them.

Theme 3: Female empowerment (Late 2000s)

One of the most crucial and visible transformations was the changing role of the female characters in Bollywood action movies. In most of the action blockbusters like Karan Arjun (1995), Jeet (1996), Soldier (1998) or Gadar (2001), the leading lady was portrayed as a damsel in distress who was ultimately saved by the male lead. However, the stereotypical portrayal of women in Bollywood saw a drastic change during the late 2000s. Action flicks like Ek Tha Tiger (2012), Singham Returns (2014), War (2019) and URI (2019) portrayed strong and self-reliant female characters, which was a rare occurrence in Bollywood action movies released during the 90s and early 2000s. Historically, women in India led a suppressed life and they were confined to taking care of their family only. However, India has been witnessing cultural and societal changes that promotes equality of opportunities to women in various domains of the society ( Krishnan, 2019). This study primarily attributes these changes to the rising education level in India. Government schemes like the Ujjwala Scheme (2007) and Beti Bachao Beti Padhao Abhyan (2015) also helped to raise the awareness of the importance of the human rights of the female population in the country. These positive changes are also reflected in most of the contemporary Bollywood movies where the role of the female lead is not just confined to being a source of solace for her male counterpart.

The increasing literacy rate, reduction in number of female infanticides, participation of women in workforce, female-friendly labour laws and workplaces, and emergence of entrepreneurial ventures from women leaders, have changed the power structure of society ( Nishi, 2021; Kaur, 2022; Hindrise 2022). Today equality is just not an abstract concept in India. It is a reality experienced by both males and females in the country which is aptly represented in movies like Ki&Ka (2016), English Vinglish (2012) and many more. The social cognitive theory of learning and unlearning ( Schunk, 2012) of certain behaviours can be utilized here as the justification of the evolving nature of society, movie genres and audience taste preferences time and again.

### Comedy

Theme 1: Rise of mainstream slapstick comedies.

The researchers found that the mainstream comedy movies became a consistent part of the top revenue grossers only after 2005. Prior to 2005, there were only two mainstream comedy movies that made it to the top five list. Post 2005, comedy films made it to the top five grossers more frequently. Interestingly, it was found that most of the popular comedies like Welcome (2007), Ready (2011), Housefull 2 (2012), Housefull 4 (2019) etc. were slapstick in nature where broad comedy, confusion and absurd situations were their prominent feature. The researchers attribute the growing popularity of slapstick comedies with the escapist mentality of the Indian audience. Escapism has been mostly associated with those individuals who seek retreat from their monotonous life ( Katz and Foulkes, 1962). The authors point out that India as a country has been gradually going down in the happiness index list. India ranks 136 among the 146 surveyed countries and its average happiness score has dropped drastically from 4.77 in 2013 to 3.82 in 2022. More than 89% of the Indian population suffer from mental stress which makes India one of the most depressed countries in the world (India Today, 2019). The change in lifestyle of the younger generation, increasing working hours and no time for physical activities and hobbies has shifted people to seek enjoyment and fulfilment through movies. Which has shown an increased indulgence towards light-hearted happy movies. Thus, the researchers assume that because of a hectic and stressful lifestyle people are drawn towards slapstick comedies in order to relieve their mental tension. Hence, Bollywood has witnessed a sharp rise in the popularity of slapstick comedies.

### Romantic comedies

Theme 1: Liberated lifestyle (after early 2000s)

On one hand, when melodramatic romantic movies saw a dip in its popularity during the early 2000s, the popularity of comedy movies was on a rise. This is when several movie makers kickstarted the era of romantic comedies in India ( Ajaz, 2019). This amalgamation of love and humour brought forward a tectonic change in the portrayal of romantic characters in Bollywood. When compared to the older romantic movies, the principal characters in rom coms like Salaam Namaste (2005), Garam Masala (2005) and Love Aaj Kal (2009) had a much more liberated view of life and romance when compared to the stereotyped Bollywood movies. The portrayal of western ideologies like live in relationships, casual dating and polyamorous relationships was popularized by rom coms. As discussed in the earlier section (see Romantic dramas), the influence of western culture was vividly visible in India. Cross-cultural communication theory explains the need of adopting western culture concepts with an Indianized blend of values. The exposure the younger generation has been through the movies, going and living abroad and social media trickles down the effect in their behaviour and choices. The younger generation found themselves more connected with the bold and liberated idea of relationships and romance which was portrayed in the contemporary rom coms.

Theme 2: Individual aspirations (after early 2000s)

This theme captures another important aspect of romantic comedies i.e., the urge of young individuals to pursue their dreams and passion instead of following the usual societal norms of pursuing a regular nine-to-five job. Several rom coms like Love Aaj Kal (2009), Ajab Prem Ki Ghazab Kahani (2009) and Yeh Jawani Hai Deewani (2013) portrayed beliefs of a progressive Indian society where every individual was ready to quit their monotonous jobs to follow their passion without any compromises. As ‘follow your passion’ became the buzz phrase of the twenty-first century (Reneau, 2021), Bollywood movie makers also started portraying characters who were independent, optimistic, and open to an alternative form of lifestyle. The researchers attribute this phenomenon with the social cognitive theory (Tyron, 2013), which provides an understanding about how people shape their behaviour and lifestyle through observational learning. It was found that the young population in India also started displaying similar attributes as they were increasingly seeking employment opportunities that aligns with their individual aspirations ( World Economic Forum, 2018). Moreover, the emergence of the entrepreneurial culture in India, gave rise to more than hundred unicorns ^2^ which provided a platform for young individuals to achieve their dreams (Invest India, 2021). Thus, the researchers theorize that as India as a country was going through an entrepreneurial wave, the audience felt more connected in watching movie characters who were willing to take risks in order to follow their passion.

## Conclusion

After thematically analysing each of the genres separately, this study proposed ten themes. However, the most interesting outcome of the study was found when the researchers looked into all the themes in conjunction. It was found that the change in the Indian audience’s preference can be broadly classified into three overarching themes which encapsulated the type of content the audiences preferred across genres for twenty-six years (see Table 3). It should be noted that the overarching theme trends depicted in Table 3 are time specific and not genre specific. They holistically capture the overall shift of the content preferred trend from 1994 – 2019 irrespective of the type of movies.

**Table 3.** Theme trends.

Themes generated from all the genres	Overarching theme trends	Timeline
Restricted access to romantic relationships (Early 90s)	Family feuds and emotional conflicts	94 – 2000s
Emotional conflicts (1990s – early 2000)		
Family revenge stories (1990s – early 2000s)		
Realistic storylines (After 2000s)	Social problems and national threats	After 2000
Addressing societal problems (After early 2000)		
Addressing national threats (Late 2000s)		
Liberated lifestyle (After early 2000s)	Growth of self-expression	After 2000
Rise of mainstream slapstick comedies (After early 2000s)		
Individual aspirations (After early 2000s)		
Female Empowerment (Late 2000s)		

It was between the 1990s and early 2000s when the Indian audience had immense affinity for watching family movies. However, with the change in the Indian family structure, the desire to watch family dramas also saw a significant dip. Not only in family dramas, but the same attribute was seen across all the genres and very rarely did movie makers focus their stories on joint families. Similarly, with the growth of the internet and rise in the awareness level of the audience, movie makers started focusing on realistic storylines and sensitive issues across several genres during the early 2000s. Social issues like drugs overdoses, rape and sexual assault, struggling education systems etc. were addressed by the movie makers. And simultaneously, it was also during the same period when the growth of self-expression among the Indian audience was strikingly evident, as they preferred watching movies which induced a sense of individuality among them. We argue that although the change in the genre preference was a gradual process and nothing changed overnight, the transformations were extremely clear and profound. The overall impact of the overarching themes was visible across all the genres. The drastic changes in the Indian society had a systematic impact on the culture of the audience which resulted in their change in preference of movie genres. Movies are nothing but a synthesized version of larger culture with respect to time and context they are developed in. It has the ability to carry strong messages to a wider audience.

From a business perspective, this study would help movie makers to understand the changing preference for movie genres in India over the last two decades and assist them in producing commercially successful movies. For example, as one can clearly observe the growth in the popularity of comedy movies, movie makers can green light more comedy scripts to tap in substantial economic benefits. They can also develop a hybrid (like romcoms) to revive a declining genre like family drama. But most importantly, the results should entice them to keep a close eye on all the major events (social, political, and cultural) that take place in India, as well as globally, to understand what excites the audience in order to produce relevant content for them. Other entertainment avenues like video games, soap operas, advertisements etc. can also pick up important cues to come up with alluring content. And although this study is focused only on the Indian subcontinent, academicians throughout the world can benefit by investigating the impact of society and culture on entertainment.

This study takes the support of three theories to explain the behaviours of the audience and creators. Traditionally, genres or storylines rest on a linear sense of temporality and contemporary cultural context ( Booth, 2012). The development of movie storylines by creators is influenced by the topical undercurrents of society, hereby revalidating the social cognitive theory of learning and unlearning, of certain behaviours in a particular timeframe. The process of making a choice by the audience, seeking certain kinds of gratification in the form of entertainment value or informational value is supported by Uses & Gratification Theory. Hence, from a theoretical perspective, this study reaffirms the relevance of the theories in the context of the Indian entertainment (movie) industry ( Bandura, 2005; Weiyan, 2015; Gross, 1985).

One of the major limitations of the study is that it excluded 25 out of the 130 selected movies. Those movies were spread across seven additional genres. As those 25 movies were inadequate to develop an adequate trend line for the analysis, they had to be dropped from the study. This resulted in the exclusion of genres like biopics, sports dramas and suspense thrillers which have become extremely popular in the recent past. For future studies, we suggest including a larger pool of movies to generate better results. Lastly, although the context of the study was limited to movies, future researchers might also make an effort to comprehend the evolutionary changes of other entertainment avenues like gaming, music, advertisement etc.

## Data Availability

Zenodo: Top 5 Bollywood grossers from 1994-2019_Zenodo, https://doi.org/10.5281/zenodo.7154229 ( Mohanty *et al*., 2022). This project contains the following underlying data:
• Top 5 Bollywood grossers from 1994-2019_Zenodo (1) Data are available under the terms of the Creative Commons Attribution 1.0 Generic (CC-BY 1.0)

## Footnotes

^1^
Anti-genre: ‘A cinema which is either free from genre or else subverts the genres of mainstream culture’ ( Jancovich, 2010).

^2^
Unicorns: a privately held startup company with a value of over $1 billion.
