# Supplementary material for: Mapping movie genre evolution (1994 – 2019) using the role of cultural and temporal shifts: a thematic analysis
Source: F1000Res. 2023 Sep 4;12:662. Originally published 2023 Jun 13. [Version 2] doi: 10.12688/f1000research.127008.2 (PMC10523098; doi:10.12688/f1000research.127008.2)
Supplement: Peer review history

# Reviewer report: Reviewer response for version 2

**DOI:** 10.5256/f1000research.155058.r203885

**Published:** 2023-09-26

**Authors:** Rebecca Bell-Metereau

**Affiliations:** 1 Department of English, Texas State University, San Marcos, Texas, USA

This article was revised to address issues I mentioned concerning the first version. It is a useful piece, and I recommend indexing.

Is the work clearly and accurately presented and does it cite the current literature?

Yes

If applicable, is the statistical analysis and its interpretation appropriate?

I cannot comment. A qualified statistician is required.

Are all the source data underlying the results available to ensure full reproducibility?

Yes

Is the study design appropriate and is the work technically sound?

Yes

Are the conclusions drawn adequately supported by the results?

Yes

Are sufficient details of methods and analysis provided to allow replication by others?

Yes

Reviewer Expertise:

NA

I confirm that I have read this submission and believe that I have an appropriate level of expertise to confirm that it is of an acceptable scientific standard.

# Reviewer report: Reviewer response for version 2

**DOI:** 10.5256/f1000research.155058.r203886

**Published:** 2023-09-25

**Authors:** Parichay Patra

**Affiliations:** 1 Department of Humanities and Social Sciences, IIT Jodhpur, Jodhpur, Rajasthan, India

I have been through the comments and changes made by the authors and am satisfied with the quality of the article. This version should be considered as ready for indexing.

Is the work clearly and accurately presented and does it cite the current literature?

Partly

If applicable, is the statistical analysis and its interpretation appropriate?

Yes

Are all the source data underlying the results available to ensure full reproducibility?

Yes

Is the study design appropriate and is the work technically sound?

Partly

Are the conclusions drawn adequately supported by the results?

Partly

Are sufficient details of methods and analysis provided to allow replication by others?

Partly

Reviewer Expertise:

NA

I confirm that I have read this submission and believe that I have an appropriate level of expertise to confirm that it is of an acceptable scientific standard.

# Reviewer report: Reviewer response for version 1

**DOI:** 10.5256/f1000research.139472.r178633

**Published:** 2023-07-04

**Authors:** Rebecca Bell-Metereau

**Affiliations:** 1 Department of English, Texas State University, San Marcos, Texas, USA

Assuming that profitability is the most important factor ignores the importance of artistic or intellectual value, which does not always correlate with a film's popularity or box office success. The author should consider the critique of Judith Hess (see “Genre films and the Status Quo,” Judith Hess, Jump Cut, no. 1, 1974, pp.1, 16, 18 copyright, Jump Cut: A Review of Contemporary Media, 1974, 2004) ^1^.

 Correct possessive in line 1, Par. 4. change "its" and "it's" to "their"

 Correct in line 4, Pr. 4. "till date" change to "to date"

 This essay does not seem to address the influence of capitalism on film, since it is a money-making and expensive medium, and it is in the interest of filmmakers to use formulaic plots and genre conventions because they are easier to produce quickly and efficiently. 

 It is also important to include consideration of the climax and resolution of films since the impression viewers have at the end of the film is an important factor in a film's success and profitability.

 The author should consider why action films are more successful, particularly for international films, because they do not rely on language for their interest and success. 

 The analysis of themes needs more sophisticated analysis. How do films encourage viewers to accept the social status quo? What about films that encourage rebellion or individualism? 

 The discussion of romantic films in conjunction with the practice of arranged marriages is fertile ground, since it dovetails with the economic aspect of the arranged marriage to maintain the practice of marrying within one's social class or improving one's social class by marrying a partner with higher status.

 In general, this is a good analysis in terms of descriptive factors. I would like to see more speculation and perhaps inclusion of discussion of those outlier films that gained international success and recognitions, to see how and why these films broke the mold and rose above the category of genre to represent original art.

Is the work clearly and accurately presented and does it cite the current literature?

Yes

If applicable, is the statistical analysis and its interpretation appropriate?

I cannot comment. A qualified statistician is required.

Are all the source data underlying the results available to ensure full reproducibility?

Yes

Is the study design appropriate and is the work technically sound?

Yes

Are the conclusions drawn adequately supported by the results?

Yes

Are sufficient details of methods and analysis provided to allow replication by others?

Yes

Reviewer Expertise:

Film and media studies, genre research, memory and film, gender and film, depictions of autism in film, international film, philosophy and feminism, auteur theory, thematic studies such as conspiracy films, environmental films, body studies, disability in film, acting, modernity in film

I confirm that I have read this submission and believe that I have an appropriate level of expertise to confirm that it is of an acceptable scientific standard, however I have significant reservations, as outlined above.

## References

[1] : Genre films and the Status Quo. *Jump Cut: A Review of Contemporary Media* .1974;1-16 Reference source

# Response

**Published:** 2023-08-23

**Authors:** Anshuman Mohanty

**Affiliations:** TAPMI, Manipal Academy of Higher Education, India

Dear Dr. Metereau,

 Thank you for giving us the opportunity to revise and resubmit our manuscript titled, ‘Mapping movie genre evolution (1994–2019) using the role of cultural and temporal shifts: a thematic analysis’ for publication. By keeping your insightful suggestions in mind, we have resubmitted the revised version of the paper.

 We have aspired to address most of the suggestions given by you in our manuscript. Below is the summary of the changes (along with the line numbers) that we made in the manuscript. All page numbers refer to the revised manuscript file with tracked changes.

***Comment 1*** **:** The first suggestion was to explore the critique of ‘Judith Hess Wright, *Genre films and the Status Quo*, Jump Cut (1974)’ to consider the fact that assuming profitability is the most important factor, it ignores the importance of artistic or intellectual value, which does not always correlate with a film's popularity or box office success.

***Response to comment 1*** **:** As suggested, we have gone through and included the aforementioned critique by Judith Hess Wright in our manuscript (line number 690-699). We have articulated that despite lacking artistic and intellectual value, genre films have always managed to achieve popularity and commercial success as they tend to provide easy comfort and solace to the audience by temporarily relieving them from real world problems.

 *Kindly note that we have cited Judith Hess Wright’s article that was published in Film Genre reader IV in 2012 (Edited by Barry Keith Grant).

***Comment 2*** **:** The second suggestion was to address grammatical errors in the manuscript.

 a. Correct possessive in line 1, Par. 4. change "its" and "it's" to "their".

 b. Correct in line 4, Pr. 4. "till date" change to "to date".

***Response to comment 2*** **:** The specific changes have been addressed in line number 47, 48 and 50.

***Comment 3*** **:** It was suggested to address the impact of capitalism on the filmmaking process which ultimately influences filmmakers to use formulaic plots and genre conventions as they are easier to produce quickly and efficiently.

***Response to comment 3*** **:** To address this comment, we have included a specific segment in our manuscript (line number 719-739) that highlights the impact of capitalism on the filmmaking process. We have pondered upon the fact that as the financial risk associated in the movie making business is growing exponentially, filmmakers are inclined to play safe by adopting formulaic plots that have a higher probability of success. We have also highlighted the adoption of the ‘ritualistic approach’ (Altman, 1984) by filmmakers to cater the needs of the audience.

***Comment 4*** **:** It was suggested to consider the climax and resolution of films since the impression viewers have at the end of the film is an important factor in a film's success and profitability.

***Response to comment 4*** **:** We have referred to Thomas Sobchack’s essay on ‘ *Genre Film: A Classical Experience*, Literature/Film Quarterly (1975)’ to address this comment (line number 685-690). We have highlighted that popular genre films have a tendency to resolve all the conflicts and ambiguities in their storyline by providing a congenial resolution at the end, which provides the audience with a feeling of catharsis that helps in boosting the popularity of the movie – hence, the commercial success. Similarly, we have also highlighted about the proclivity of Bollywood filmmakers to provide an ending that instils a sense of satisfaction and harmony among the audience (line number 699-711).

* Kindly note that we have cited Thomas Sobchack’s article that was published in Film Genre reader IV in 2012 (Edited by Barry Keith Grant).

***Comment 5*** **:** It was suggested to highlight why international Action films have become more successful despite not relying on language for success.

***Response to comment 5*** **:** We have used the concept of ‘cultural discount’ in order to explain the global acceptance of international action films (line number 510-520). This concept states that a cultural product (movie) rooted in one culture will have a diminished appeal elsewhere as viewers might find it difficult to identify with the values and beliefs of the foreign culture. However, as action films rely more on graphic violence, they have a universalistic appeal and are resistant to cultural discount when compared to other genres like comedy or drama which are more culturally specific.

***Comment 6*** **:** The sixth comment was divided into two parts.

**a. **It was suggested to highlight how movies encourage viewers to accept social status quo.

**b.** And it was also suggested to analyze movies those have encouraged a sense of individualism and rebellion among the audience.

***Response to comment 6a*** **:** In order to address this comment, we have referred to Judith Hess Wright’s essay on *‘Genre films and the Status Quo’*. In our write-up we have highlighted that popular genre films helps to maintain social status quo as these films helps the audience to temporarily transport to an imaginative narrative where all problems have simplistic solutions (line number 693-696). The explanation is in line with the prevalence of a congenial climax/resolution that satisfies the audience at the end of the movie.

***Response to comment 6b*** **:** We have considered Bellah et al.’s (1996) proposition of ‘expressive individualism’ to highlight movies that encouraged an individualistic and rebellious way of life (line number 638-650). We have explained how several characters in romantic comedies portrayed an eccentric lifestyle that aligned with the contemporary individualistic ideology of the west.

***Comment 7*** **:** It was suggested to include the discussion of outlier movies that gained international success and recognitions, to see how and why these films broke the mold and rose above the category of genre to represent original art.

***Response to comment 7*** **:** As suggested, we have attempted to discuss about original art movies (parallel cinema) in our manuscript that does not follow the narrative conventions of a popular genre film (line number 768-796). We have cited examples of several internationally acclaimed Bollywood art films that touched upon sensitive topics with high level of realism in their storylines. We have speculated that despite receiving international recognition, art movies are not commercially successful in the domestic market as they do not provide a congenial resolution that helps in maintaining social status quo among the audience. However, we would like to point out that our analysis has focused only on the popular genre movies in India and thus, we have suggested that an in-depth analysis of art cinemas in future can provide detailed insights on the socio-political state of the country.

 We would like to thank you for your time and effort in reviewing our manuscript. The feedback has been invaluable in improving the content and presentation of the paper. We look forward to hearing from you.

 Thanks.

 Regards.

# Reviewer report: Reviewer response for version 1

**DOI:** 10.5256/f1000research.139472.r178632

**Published:** 2023-06-28

**Authors:** Parichay Patra

**Affiliations:** 1 Department of Humanities and Social Sciences, IIT Jodhpur, Jodhpur, Rajasthan, India

This article has its promises, primarily because of its interdisciplinary appeal. Here a number of management scholars are using a fairly large dataset and statistical methods to offer their comments on an apparently specific trend of cinema-going and film business in India. While their interest in this mode of industrial research with cinema as their site of inquiry seems rewarding, it should also be noted that the understanding and interpretation of genre requires more careful consideration of existing literature. 

 Allow me to elaborate on this. How to identify a genre? In the context of the global north, there are semantic and syntactic methods for that (please refer to Rick Altman’s iconic essay). In the global south, it’s not that easy. Indian cinema scholars have often argued that the identification of genres in the context of South Asia/India is a challenge as, unlike Hollywood, Bombay popular cinema does not have clearly demarcating genres. The presence of the ‘social’ as an all-encompassing super-genre in Bombay cinema has been theorized by M. Madhava Prasad, who also went on to suggest that genre-mixing works as ‘creative fabrication’ across several Indian cine-industries. 

 The article does not refer to such context-specific theorizations and methods. Instead, despite its methodological promises, it often resorts to generalizations without considering the temporal and ideological differences between films. For instance, it groups *Hamara Dil Aapke Paas Hai *(2000) and *Toilet: Ek Prem Katha* (2017) under the same section, contextualizing them in a similar way, ignoring the enormous temporal gap between them. 

 Moreover, the ideological transitions have not been taken into account. Films such as *Toilet: Ek Prem Katha* and *Uri: The Surgical Strike* (2019) belong unquestionably to the ‘new India’ of the Narendra Modi regime and its cine-politics, with the Prime Minister praising the former for its ‘message’ and the latter playing a significant role in the pre-2019 election Indian public sphere. *Uri*’s success led to the making of a series of propaganda films favored actively by the present regime, many of them becoming major hits through their controversies. 

 The article needs major revision, references to existing literature should be made, and several generalizations might be avoided.

Is the work clearly and accurately presented and does it cite the current literature?

Partly

If applicable, is the statistical analysis and its interpretation appropriate?

Yes

Are all the source data underlying the results available to ensure full reproducibility?

Yes

Is the study design appropriate and is the work technically sound?

Partly

Are the conclusions drawn adequately supported by the results?

Partly

Are sufficient details of methods and analysis provided to allow replication by others?

Partly

Reviewer Expertise:

Film history, film aesthetics

I confirm that I have read this submission and believe that I have an appropriate level of expertise to confirm that it is of an acceptable scientific standard, however I have significant reservations, as outlined above.

# Response

**Published:** 2023-08-23

**Authors:** Anshuman Mohanty

**Affiliations:** TAPMI, Manipal Academy of Higher Education, India

Dear Dr. Patra,

 Thank you for giving us the opportunity to revise and resubmit our manuscript. By keeping your insightful suggestions in mind, we have resubmitted the revised version of the paper titled, ‘Mapping movie genre evolution (1994–2019) using the role of cultural and temporal shifts: a thematic analysis’ for publication.

 We have aspired to address most of the suggestions given by you in our manuscript. Below is the summary of the changes (along with the line numbers) that we made in the manuscript. All page numbers refer to the revised manuscript file with tracked changes.

***Comment 1*:** The first suggestion was to explore context specific theorizations on movie genres laid down by well-known researchers like Rick Altman and M. Madhava Prasad to comprehend the interpretation of genre in the global north and global south. It was also suggested to look into the presence of the ‘all-encompassing social genre’ and ‘genre mixing’ in India.

***Response to comment number 1:*** As suggested, we have included Rick Altman’s iconic essay on semantic and syntactic genre in the literature review section of our manuscript (line number 95-114). Then we have focused on M. Madhava Prasad’s (2011) paper, where he has mentioned about the presence of the all-encompassing social genre in India post the independence era (line number 115-132).

 However, we have also cross referred to Ravi Vasudevan’s book ‘ *The melodramatic public*’ to emphasize the rise of the ‘self-conscious genre cinema’ in India (line number 133-177). Referring to Vasudevan’s work, Prasad (2011) has also acknowledged the emergence of the self-conscious genre in India from the 1990s. In his book, Vasudevan has explained how movie making was revolutionised after the economic liberalisation in the 1990s which gave rise to a contemporary film making style in India. He also attributes this change to the corporatisation of the film industry which supported several small players (film institute graduates and theatre professionals) to produce movies which was inspired by Hollywood genres in terms of narrative integration. Thus, although we acknowledge the presence of the ‘social’ genre as explained by Prasad, we cannot overlook the rise of the self-conscious genres in India during 1990s. And as we have analysed movies released after 1994, a phase which witnessed the growth of the self-conscious genres, we have interpreted movies based on specific genres rather than identifying them as ‘socials’ which was regarded as a ‘superficial’ label as it did not do justice to most of the movies to which it was applied to (Prasad 2011 and Vasudevan, 2011). Secondly, although we acknowledge the phenomenon of ‘genre mixing’ as explained by Prasad, we have resorted to categorise movie genres based on the core-content (theme) of their storyline (line number 177-199). In the revised manuscript we have stated that although majority of the popular cinemas in India are typically hybrids (inspired from several genres), the story line revolves around a specific theme/topic. Thus, we have interpreted movie genres based on the core-content portrayed in their narratives.

***Comment 2*: **The second suggestion was to avoid generalizations while citing examples of movies that does not consider the temporal differences between films.

***Response to comment 2*: **As rightly suggested by you, we have tried to avoid several generalizations while citing movie examples in the manuscript. For example, we have removed the grouping of certain films like Humara Dil Apke Paas Hai (line number 452), Kabhi Khusi Kabhi Gham (line number 477), Kya Kehna (line number 485) and Wanted and Dabanng (line number 538). We have attempted to cite examples of movies which have certain commonalities between them without tampering the temporal gap.

***Comment 3*: **The third suggestion was to consider the ideological transitions in cine politics that has impacted the way in which movies are being made in the present time. We were told to explore how the present government’s ideology of ‘new India’ has been embedded in the narratives of several movies.

***Response to comment 3*:** We have included specific examples of movies under the explanation of ‘Romantic Drama’ (line number 445-450) and ‘Action’ (line number 547-558) genre in our analysis that highlights the overt appreciation of the present government’s ideology of the ‘new India’ which has been focusing a lot on the importance of health and hygiene, make in India products etc. We have also touched upon the present government’s affinity for pro-nationalistic themes in their narratives. We have aspired to convey that movies supporting the present regime’s ideology gets active political support from the government which ultimately boosts their popularity. However, I would like to point out that although several movies like The Kashmir Files (2022), The Kerala Story (2023) etc. gained immense success owing to their pro-nationalistic themes and controversial storylines, they have not been taken into consideration as they were released after 2019 which is beyond the scope of our analysis.

 We would like to thank you for your time and effort in reviewing our manuscript. The feedback has been invaluable in improving the content and presentation of the paper. We look forward to hearing from you.

 Thanks.

 Regards.
